# Magnetic properties of solid solutions between BiCrO_3_ and BiGaO_3_ with perovskite structures

**DOI:** 10.1088/1468-6996/16/2/026003

**Published:** 2015-04-28

**Authors:** Alexei A Belik

**Affiliations:** International Center for Materials Nanoarchitectonics (WPI-MANA), National Institute for Materials Science (NIMS), 1-1 Namiki, Tsukuba, Ibaraki 305-0044, Japan

**Keywords:** perovskites, multiferroics, high-pressure, BiCrO_3_, magnetic properties

## Abstract

Magnetic properties of BiCr_1−*x*_Ga_*x*_O_3_ perovskite-type solid solutions are reported, and a magnetic phase diagram is established. As-synthesized BiCrO_3_ and BiCr_0.9_Ga_0.1_O_3_ crystallize in a monoclinic (*m*) C2/c structure. The Néel temperature (*T*_N_) decreases from 111 K in BiCrO_3_ to 98 K in BiCr_0.9_Ga_0.1_O_3_, and spin-reorientation transition temperature increases from 72 K in BiCrO_3_ to 83 K in BiCr_0.9_Ga_0.1_O_3_. *o*-BiCr_0.9_Ga_0.1_O_3_ with a PbZrO_3_-type orthorhombic structure is obtained by heating *m*-BiCr_0.9_Ga_0.1_O_3_ up to 573 K in air; it shows similar magnetic properties with those of *m*-BiCr_0.9_Ga_0.1_O_3_. *T*_N_ of BiCr_0.8_Ga_0.2_O_3_ is 81 K, and *T*_N_ of BiCr_0.7_Ga_0.3_O_3_ is 63 K. Samples with *x* = 0.4, 0.5, 0.6 and 0.7 crystallize in a polar R3c structure. Long-range antiferromagnetic order with weak ferromagnetism is observed below *T*_N_ = 56 K in BiCr_0.6_Ga_0.4_O_3_, *T*_N_ = 36 K in BiCr_0.5_Ga_0.5_O_3_ and *T*_N_ = 18 K in BiCr_0.4_Ga_0.6_O_3_. BiCr_0.3_Ga_0.7_O_3_ shows a paramagnetic behaviour because the Cr concentration is below the percolation threshold of 31%.

## 1. Introduction

Bi-containing perovskites have received a lot of attention as multiferroic materials and lead-free ferroelectrics [1–4]. The stereochemically active 6s^2^ lone pair of a Bi^3+^ ion plays an important role in producing polar distortions in Bi-containing perovskites. They form a basis for materials with super-tetragonality and a huge spontaneous polarization (*P*_S_) observed, for example, in BiCoO_3_ [4], Bi_2_ZnTiO_6_ [5], Bi_2_ZnVO_6_ [6], BiCo_0.3_Fe_0.7_O_3_ [7], and highly strained BiFeO_3_ thin films [3, 8]. Bulk BiFeO_3_ [3] and BiAlO_3_ [4] also crystallize in a polar structure with space group R3c and large *P*_S_.

On the other hand, BiCrO_3_ [9, 10] crystallizes in a centrosymmetric structure with space group C2/c [4, 11–13]. There is a structural phase transition to a GdFeO_3_-type Pnma structure from 420 K in BiCrO_3_ [9–12]. It is interesting that during the Pnma-to-C2/c phase transition, twin domains with the size of less than 10 nm are formed [12, 14]. First-principle calculations could not explain the experimental C2/c structure and predicted the GdFeO_3_-type Pnma structure as the ground-state structure [15, 16]. The Néel temperature (*T*_N_) of BiCrO_3_ is 109–111 K [9–13, 17], and there is a spin-reorientation transition below 75–80 K in BiCrO_3_ [12, 13, 17].

Among Bi*M*O_3_ (*M* = 3d transition metals) compounds, BiCrO_3_ is one of the least studied compounds, especially its solid solutions with isovalent substitutions. From the BiCrO_3_-rich side, just a few selected compositions have been investigated in Bi_1−*x*_Y_*x*_CrO_3_ (*x* = 0.01, 0.05, 0.2 and 0.5 [13] and 0.1 [18]), BiCr_1−*x*_Fe_*x*_O_3_ (*x* = 0.5 [19]) and BiCr_1−*x*_Mn_*x*_O_3_ (*x* = 0.5 [20]) bulk systems. We have recently identified BiGaO_3_-based perovskites, Bi*M*_1−*x*_Ga_*x*_O_3_ (*M* = Cr, Mn and Fe), as a large family of polar materials, which includes phases with (pseudo) super-tetragonality and R3c symmetry [21]. In particular, the R3c polar phase was found at 0.4 ≤ *x* ≤ 0.7 in BiCr_1−*x*_Ga_*x*_O_3_ solid solutions even though the end members, BiCrO_3_ [4, 11] and BiGaO_3_ [4], crystallize in centrosymmetric crystal structures. In this work, we report on detailed magnetic properties of the BiCr_1−*x*_Ga_*x*_O_3_ solid solutions.

## 2. Experimental section

BiCr_1−*x*_Ga_*x*_O_3_ with *x* = 0, 0.1, 0.2, 0.3, 0.4, 0.5, 0.6 and 0.7 were prepared from stoichiometric mixtures of Bi_2_O_3_ (99.9999%), Cr_2_O_3_ (99.99%) and Ga_2_O_3_ (99.99%). The mixtures were reground under acetone several times, placed in Pt capsules, dried at 573 K for several days and finally treated in a belt-type high-pressure apparatus at 6 GPa and 1700 K for 2 h (heating rate to the desired temperature was 10–15 min). After heat treatment, the samples were quenched to room temperature (RT), and the pressure was slowly released. Samples with other *x* values were mixtures of two perovskite phases (R3c and Cm phases for *x* = 0.8) and perovskite (Cm) and pyroxene phases for *x* = 0.9 [21]; therefore, their magnetic properties were not studied.

X-ray powder diffraction (XRPD) data were collected at RT on a RIGAKU Ultima III diffractometer using CuK_*α*_ radiation (2*θ* range of 10°–120°, a step width of 0.02°, and a counting time of 2–14 s/step). The samples contained small amounts of Cr_2_O_3_ and Bi_2_O_2_CO_3_ impurities. The formation of Bi_2_O_2_CO_3_ impurity was observed in many other works [13], and it is usually attributed to the diffusion of carbon from carbon heaters through cracks in capsules. Cr_2_O_3_ impurity could remain because a small amount of Bi_2_O_3_ was removed as Bi_2_O_2_CO_3_ from stoichiometric mixtures.

Magnetic susceptibilities (*χ* = *M*/*H*) were measured using pellets on SQUID magnetometers (Quantum Design, MPMS XL and 1 T) between 2–5 and 300–400 K in different applied fields. Samples were rapidly inserted into magnetometers kept at 10 K and having a zero magnetic field; then, temperature was set to 2 or 5 K; at 2 or 5 K, measurement magnetic fields were applied; and finally measurements were performed on heating up to 300, 350, or 400 K. This procedure gave zero-field-cooled (ZFC) curves. After ZFC measurements, samples were measured on cooling resulting in field-cooled (FC) curves. Isothermal magnetization measurements were performed between −50 and 50 kOe or between −10 and 10 kOe at different temperatures. Frequency dependent ac susceptibility measurements at a zero static magnetic field were performed with a Quantum Design MPMS 1 T instrument from 150 to 2 K at frequencies (*f*) of 2, 110, and 300 Hz and an applied oscillating magnetic field (*H*_ac_) of 5 Oe. Specific heat, *C*_p_, at a zero magnetic field was recorded between 2 and 300 K on cooling by a pulse relaxation method using a commercial calorimeter (Quantum Design PPMS).

Differential scanning calorimetry (DSC) curves were recorded on a Mettler Toledo DSC1 STAR^e^ system at a heating/cooling rate of 10 K min^−1^ from 290 to 573 K in open aluminium capsules; three cycles were performed to check the reproducibility.

## 3. Results and discussion

**Figure 1.**
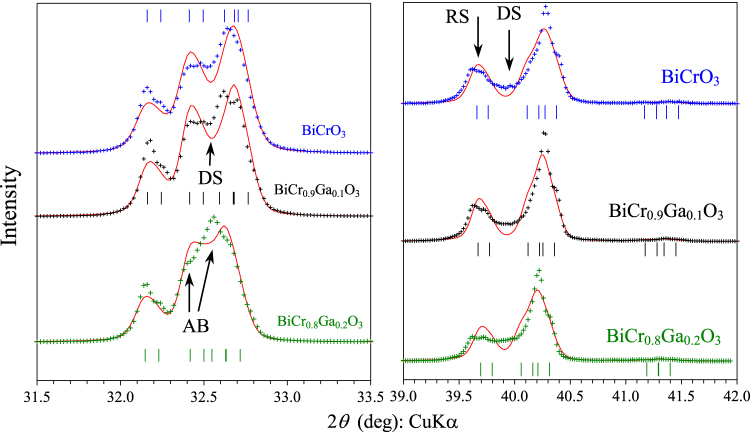
Portions of experimental (crosses) and calculated (lines) x-ray powder diffraction patterns of as-synthesized BiCrO_3_, *m*-BiCr_0.9_Ga_0.1_O_3_ and *m*-BiCr_0.8_Ga_0.2_O_3_ measured with the CuK*α* radiation at room temperature. Possible Bragg positions of the C2/c structure are indicated by tick marks. ‘AB’ means reflections with anisotropic broadening, ‘DS’ means diffuse scattering between some reflections, and ‘RS’ means reflection shifts from their ideal expected positions.

As-synthesized BiCrO_3_ and *m*-BiCr_0.9_Ga_0.1_O_3_ crystallize in the monoclinic (*m*) C2/c structure. The lattice parameters refined by the Rietveld method are *a* = 9.4786(4) Å, *b* = 5.4852(2) Å, *c* = 9.5824(4) Å and *β* = 108.587(3)° for BiCrO_3_ and *a* = 9.4762(4) Å, *b* = 5.4899(2) Å, *c* = 9.5791(4) Å and *β* = 108.571(3)° for *m*-BiCr_0.9_Ga_0.1_O_3_. However, both BiCrO_3_ and BiCr_0.9_Ga_0.1_O_3_ show noticeable anisotropic broadening (AB) of some reflections, the appearance of continuous diffuse scattering (DS) between some reflections, and shifts of some reflections (RS) from their ideal/expected positions (figure 1). Those features are most probably originated from local disorder, the presence of nanodomains [12, 14] and high concentration of domain boundaries and defects; they make the precise Rietveld analysis impossible and result in large variations in refined lattice parameters depending on models used. Similar features are observed in as-synthesized *m*-BiCr_0.8_Ga_0.2_O_3_ and *m*-BiCr_0.7_Ga_0.3_O_3_ (figures S1–S3 of the electronic supporting information (ESI)). If those features are modelled as a second perovskite phase (a PbZrO_3_-related phase with space group Pnma [21]) in the Rietveld analysis, the weight fraction of the second phase is estimated to be about 20% in BiCrO_3_, 20% in *m*-BiCr_0.9_Ga_0.1_O_3_, 30% in *m*-BiCr_0.8_Ga_0.2_O_3_ and 50% in *m*-BiCr_0.7_Ga_0.3_O_3_ [21]. Electron microscopy and electron diffraction studies showed that the AB, DS and RS features are intrinsic for BiCrO_3_ and do not originate from the presence of other perovskite phases [12, 14]. They can also be considered as intrinsic in *m*-BiCr_0.9_Ga_0.1_O_3_ based on the above numbers. However, for unambiguous interpretation of those features in *m*-BiCr_0.8_Ga_0.2_O_3_ and *m*-BiCr_0.7_Ga_0.3_O_3_ detailed high-resolution electron microscopy studies are needed.

*m*-BiCr_0.9_Ga_0.1_O_3_ exhibits a structural phase transition and shows a peak on the DSC curve at about 500 K on heating, and at 480 K on cooling. After heating as-synthesized *m*-BiCr_0.9_Ga_0.1_O_3_ up to 573 K, its XRD pattern changes. All fundamental perovskite reflections remain the same; however, weak superstructure reflections change. Reflections can be indexed in the PbZrO_3_-related structure [21] with space group Pnma and lattice parameters of *a* = 5.4892(4) Å, *b* = 15.4761(9) Å and *c* = 11.1269(7) Å (figure 3); this sample will be called *o*-BiCr_0.9_Ga_0.1_O_3_. Irreversible transformations of high-pressure phases were also found, for example, in BiFe_0.5_Sc_0.5_O_3_ [22]. The origin of the irreversible behaviour is the existence of competing phases.[Fig F2]

**Figure 2. F2:**
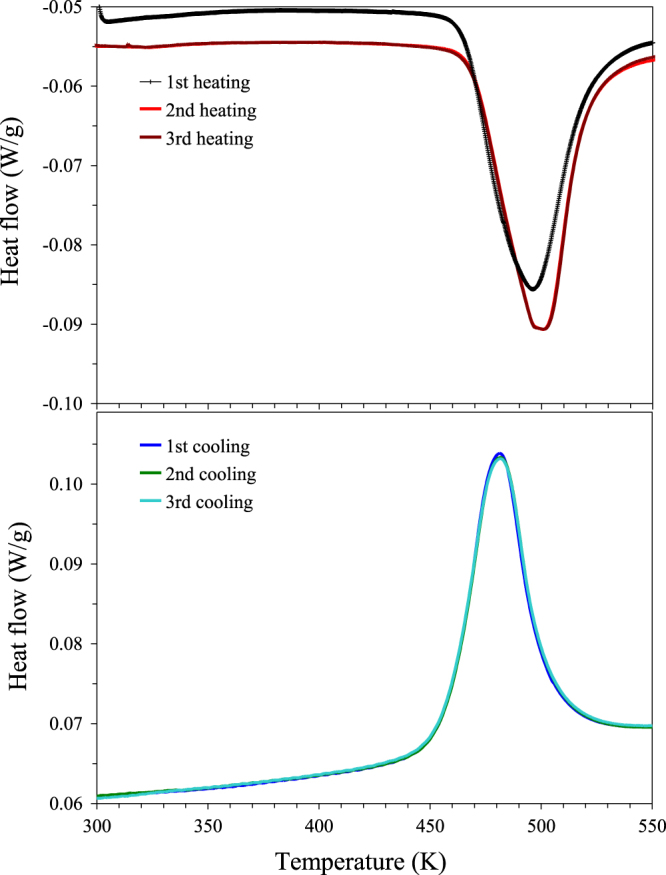
Differential scanning calorimetry curves of BiCr_0.9_Ga_0.1_O_3_ measured at a heating/cooling rate of 10 K min^−1^ between 290 and 573 K. Results of three heating–cooling cycles are shown.

**Figure 3.**
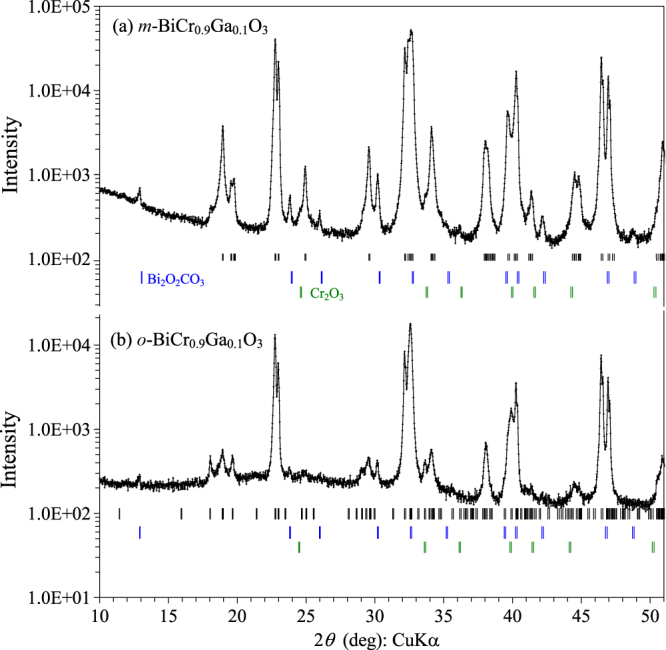
Portions of experimental x-ray powder diffraction patterns of (a) *m*-BiCr_0.9_Ga_0.1_O_3_ and (b) *o*-BiCr_0.9_Ga_0.1_O_3_ measured with the CuK*α* radiation at room temperature in the logarithmic scale to emphasize the difference in weak reflections. Possible Bragg positions are indicated by tick marks for the main perovskite phases and Bi_2_O_2_CO_3_ and Cr_2_O_3_ impurities.

**Figure 4.**
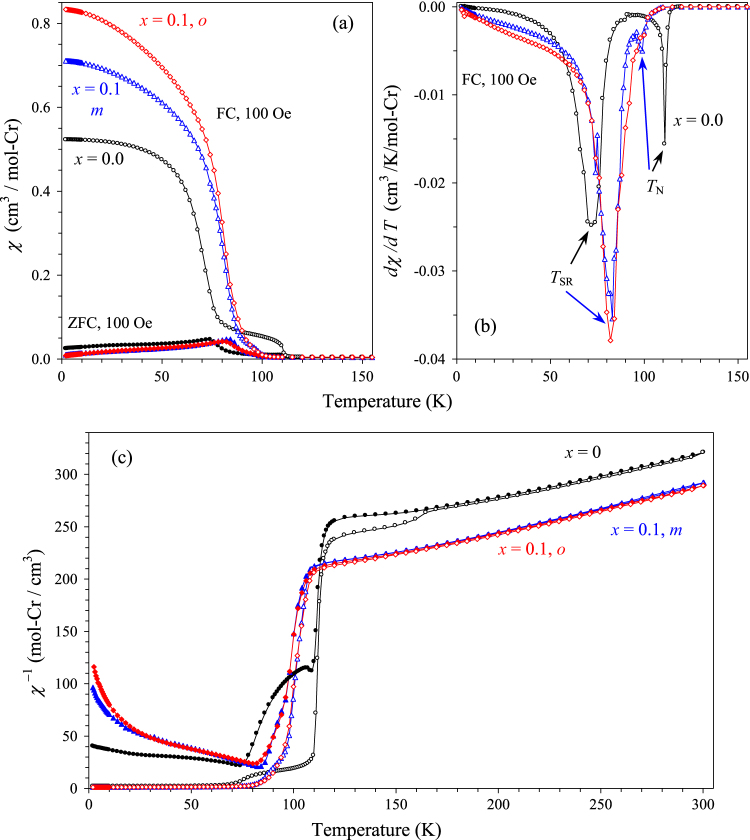
Magnetic properties of BiCr_1−*x*_Ga_*x*_O_3_ (*x* = 0, *m*—0.1 and *o*—0.1). (a) ZFC (filled symbols) and FC (empty symbols) dc magnetic susceptibility curves measured at 100 Oe. (b) FC d*χ*/d*T* versus *T* curves at 100 Oe; peaks define phase transition temperatures. (c) ZFC and FC *χ*^−1^ versus *T* curves measured at 100 Oe.

**Figure 5.**
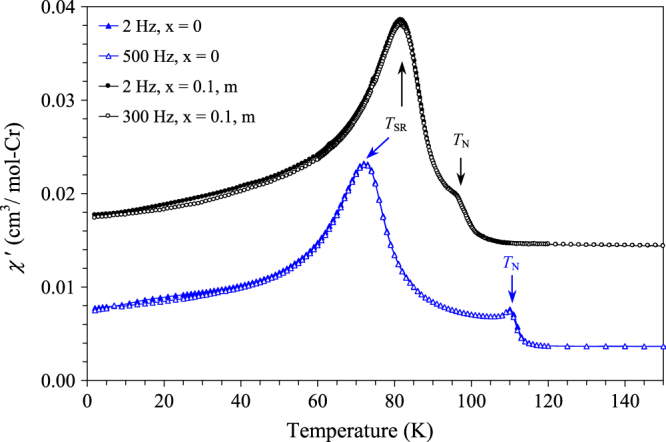
Real parts of the ac susceptibility curves of BiCr_1−*x*_Ga_*x*_O_3_ (*x* = 0 [17] and *m*—0.1). *T*_N_ is the Néel temperature, and *T*_SR_ is temperature of a spin-reorientation transition. The data for *m*-BiCr_0.9_Ga_0.1_O_3_ are shifted by +0.01 cm^3^/mol-Cr for the clarity.

BiCrO_3_ has the Néel temperature (*T*_N_) of 111 K (determined/defined here by peak positions on the FC d*χ*/d*T* versus *T* curves at 100 Oe). Below *T*_N_, the magnetic moments of Cr^3+^ ions in BiCrO_3_ are aligned along the *a* axis [14] in the G-type antiferromagnetic (AFM) structure, where all Cr–O–Cr interactions are AFM. BiCrO_3_ has a spin-reorientation transition (*T*_SR_) at 72 K (also defined by peak positions on the FC d*χ*/d*T* versus *T* curves at 100 Oe), where Cr^3+^ spins start to rotate away from the *a* axis in the (*a*, *c*) plane [13], but keeping the G-type AFM arrangement. Characteristic anomalies at *T*_N_ and *T*_SR_ can be clearly seen on the *χ* versus *T*, d*χ*/d*T* versus *T* and *χ*′ versus *T* curves of BiCrO_3_ and *m*-BiCr_0.9_Ga_0.1_O_3_ (figures 4(a), (b) and 5). We note that small anomalies are observed at 165 K in BiCrO_3_ on the 100 Oe FC *χ*^−1^ versus *T* curve (figure 4(c)); they were suggested to originate from a very small amount of the GdFeO_3_-type Pnma modification of BiCrO_3_ [4, 18]. We note that those anomalies at 165 K with different magnitudes were observed in all checked BiCrO_3_ samples (about a dozen of different samples (figure S17 of ESI), even synthesized by different groups); and those anomalies cannot be eliminated by further annealing and very slow cooling [18] suggesting that they are ‘intrinsic’ for bulk BiCrO_3_ samples. As-synthesized *m*-BiCr_0.9_Ga_0.1_O_3_ shows very similar magnetic behaviour with that of BiCrO_3_, but with *T*_N_ = 98 K and *T*_SR_ = 83 K. No additional magnetic anomalies above *T*_N_ are found in *m*-BiCr_0.9_Ga_0.1_O_3_ in comparison with BiCrO_3_; it can be related with the higher temperature of the C2/c-to-Pnma transition in BiCr_0.9_Ga_0.1_O_3_ that results in a complete transformation. Magnetic properties of *m*-BiCr_0.9_Ga_0.1_O_3_ and *o*-BiCr_0.9_Ga_0.1_O_3_ are very similar with each other (figures 4 and S13 of ESI). Inverse magnetic susceptibilities of BiCrO_3_, *m*-BiCr_0.9_Ga_0.1_O_3_ and *o*-BiCr_0.9_Ga_0.1_O_3_ are given on figure 4(c). They show a noticeable deviation from the Curie–Weiss behaviour far above *T*_N_; this is why the Curie–Weiss fits are performed above 250 K for those samples (table 1). This fact can also explain why the effective magnetic moments are slightly larger than expected ones.

**Table 1.** Temperatures of intrinsic magnetic transitions and results of the Curie–Weiss fits for as-synthesized BiCr_1−*x*_Ga_*x*_O_3_.

Sample	*T*_N_ (K)	*T*_SR_ (K)	*μ*_eff_ per Cr^3+^	*θ*
BiCrO_3_	111	72	4.161(14)*μ*_B_	−351(5) K
*m*-BiCr_0.9_Ga_0.1_O_3_	98	83	4.069(10)*μ*_B_	−282(3) K
*m*-BiCr_0.8_Ga_0.2_O_3_	81		3.965(4)*μ*_B_	−239(1) K
*m*-BiCr_0.7_Ga_0.3_O_3_	63		4.116(10)*μ*_B_	−239(3) K
BiCr_0.6_Ga_0.4_O_3_	56		3.839(3)*μ*_B_	−148.6(6) K
BiCr_0.5_Ga_0.5_O_3_	36		3.884(1)*μ*_B_	−119.8(2) K
BiCr_0.4_Ga_0.6_O_3_	18		3.889(2)*μ*_B_	−96.3(3) K
BiCr_0.3_Ga_0.7_O_3_	No		3.828(3)*μ*_B_	−73.3(5) K

*μ*_eff_ is an effective magnetic moment, *θ* is the Curie–Weiss temperature. The calculated *μ*_eff_ for Cr^3+^ (*S* = 3/2) is 3.87*μ*_B_. For the Curie–Weiss fits, the FC curves at 10 kOe are used, and the data are corrected for diamagnetic contributions from sample holders and core diamagnetism. The Curie–Weiss fits are performed between 250 and 400 K for *x* = 0 and 0.1, 250 and 340 K for *x* = 0.2 and 0.3 and 150 and 400 K for *x* = 0.4–0.7 (figure S9 of the ESI).

**Figure 6.**
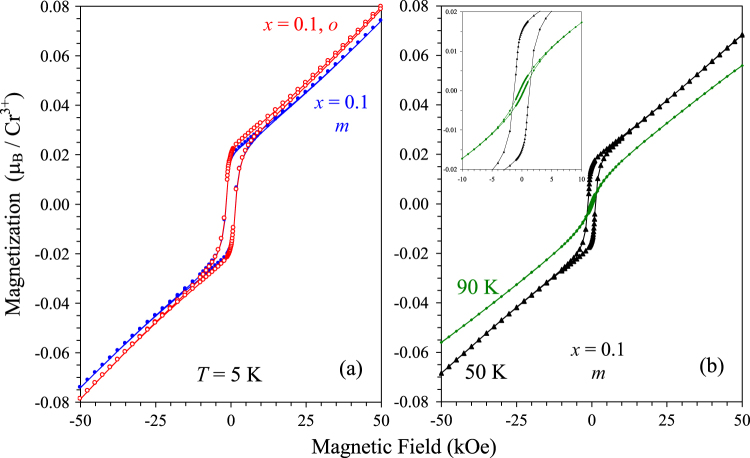
(a) Isothermal magnetization curves of *m*-BiCr_0.9_Ga_0.1_O_3_ and *o*-BiCr_0.9_Ga_0.1_O_3_ at 5 K. (b) Isothermal magnetization curves of *m*-BiCr_0.9_Ga_0.1_O_3_ at 50 and 90 K. Insert shows details near the origin.

Isothermal magnetization curves of BiCr_0.9_Ga_0.1_O_3_ are given on figure 6. They show that a very weak ferromagnetic moment is developed below *T*_N_ in *m*-BiCr_0.9_Ga_0.1_O_3_; below *T*_SR_, the hysteresis loop becomes more defined. The magnetization reaches 0.074*μ*_B_/Cr at 5 K and 50 kOe; at 5 K, the remnant magnetization is 0.0177*μ*_B_/Cr, and the coercitive field is about 1.8 kOe in *m*-BiCr_0.9_Ga_0.1_O_3_. Very similar *M* versus *H* curves were obtained in BiCrO_3_ [17]. Spin canting angles in BiCrO_3_ could not be determined from neutron diffraction because of their small values [12, 13].

Detailed magnetic properties (*χ* versus *T*, *χ*^−1^ versus *T*, d*χ*/d*T* versus *T*, *χ*′ versus *T*, *χ*″ versus *T*, *M* versus *H* and *C*_p_/*T* versus *T*) of as-synthesized *m*-BiCr_0.8_Ga_0.2_O_3_ and *m*-BiCr_0.7_Ga_0.3_O_3_ are given in figures S8–S11 of the ESI. *T*_N_ is found to be 81 K in BiCr_0.8_Ga_0.2_O_3_ and 63 K in BiCr_0.7_Ga_0.3_O_3_ from d*χ*/d*T* versus *T* and *C*_p_/*T* versus *T* curves. *T*_N_ remains the same (figure S13 of ESI) for the as-synthesized samples and samples after DSC experiments (up to 623 K for BiCr_0.8_Ga_0.2_O_3_ and 773 K for BiCr_0.7_Ga_0.3_O_3_ [21]). Magnetic entropy is almost independent of *x* for 0.0 ≤ *x* ≤ 0.3 and varies between 5.2 and 5.5 J K^−1^mol^−1^ (figure S14 of the ESI).

**Figure 7.**
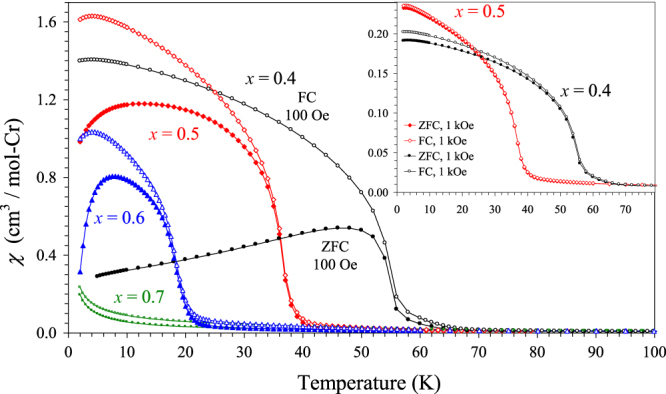
ZFC (filled symbols) and FC (empty symbols) dc magnetic susceptibility curves of BiCr_1−*x*_Ga_*x*_O_3_ (R3c) (*x* = 0.4, 0.5, 0.6 and 0.7) measured at 100 Oe. The insert gives the ZFC and FC *χ* versus *T* curves for *x* = 0.4 and 0.5 measured at 1 kOe.

**Figure 8.**
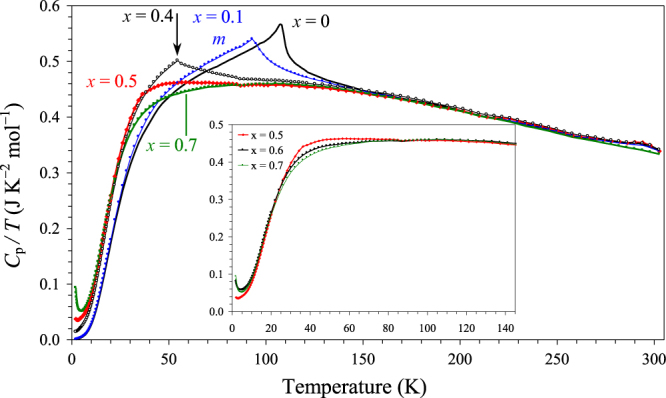
Specific heat data of BiCr_1−*x*_Ga_*x*_O_3_ at 0 Oe plotted as *C*_p_/*T* versus *T*. The vertical arrow shows the Néel temperature (*T*_N_) of BiCr_0.6_Ga_0.4_O_3_. The insert gives a fragment of the *C*_p_/*T* versus *T* curves of BiCr_0.5_Ga_0.5_O_3_, BiCr_0.4_Ga_0.6_O_3_ and BiCr_0.3_Ga_0.7_O_3_.

**Figure 9.**
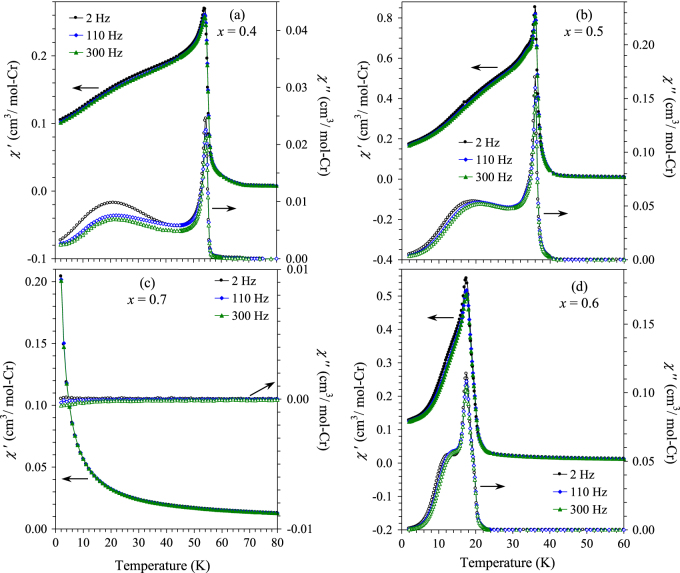
Real (*χ*′ versus *T*) and imaginary (*χ*″ versus *T*) parts of the ac susceptibility curves of BiCr_1−*x*_Ga_*x*_O_3_ (R3c) with *x* = (a) 0.4, (b) 0.5, (c) 0.7 and (d) 0.6.

The *χ* versus *T* curves of BiCr_1−*x*_Ga_*x*_O_3_ with *x* = 0.4, 0.5, 0.6 and 0.7 having the R3c polar symmetry are shown on figure 7, and the parameters of the Curie–Weiss fits are summarized in table 1 (the fits are given in figure S9 of the ESI). Effective magnetic moments per a Cr^3+^ ion are close to the expected value for those samples. The temperature of magnetic transitions in BiCr_1−*x*_Ga_*x*_O_3_ monotonically decreases with increasing *x* indicating that the magnetic transitions are intrinsic. The ZFC and FC curves of BiCr_0.6_Ga_0.4_O_3_ are typical for canted antiferromagnets; in particular, the FC curves demonstrate the saturation behaviour. In addition, BiCr_0.6_Ga_0.4_O_3_ shows a weak specific heat anomaly at 55 K (figure 8). These features indicate that there is a long-range magnetic ordering in BiCr_0.6_Ga_0.4_O_3_. The *χ*′ versus *T* and *χ*″ versus *T* curves of BiCr_0.6_Ga_0.4_O_3_ (figure 9(a)) show sharp and frequency-independent peaks at *T*_N_ with additional very broad anomalies near 22 K (on the *χ*″ versus *T* curves).

No characteristic *λ*-type anomaly is observed on specific heat of BiCr_0.5_Ga_0.5_O_3_. But we observe an excess of magnetic specific heat between about 20 and 80 K in BiCr_0.5_Ga_0.5_O_3_ in comparison with BiCr_0.3_Ga_0.7_O_3_ (figures 8 and S15 of the ESI), which comes from magnetic interactions between Cr^3+^ ions. A low temperature, the tail on the specific heat increases with increasing *x* in BiCr_1−*x*_Ga_*x*_O_3_ probably from short-range magnetic interactions. Despite the absence of specific heat anomalies, magnetic properties of BiCr_0.5_Ga_0.5_O_3_ and BiCr_0.4_Ga_0.6_O_3_ are very similar with those of BiCr_0.6_Ga_0.4_O_3_, but with transitions at lower temperatures of 36 and 18 K, respectively. In particular, the *χ*′ versus *T* and *χ*″ versus *T* curves of BiCr_1−*x*_Ga_*x*_O_3_ with *x* = 0.6, 0.5 and 0.4 are remarkably similar to each other (figure 9) demonstrating sharp and frequency-independent anomalies at *T*_N_. Therefore, we can assume that BiCr_0.5_Ga_0.5_O_3_ and BiCr_0.4_Ga_0.6_O_3_ also have long-range magnetic ordering at *T*_N_ = 36 and 18 K, respectively. Because of the highly diluted magnetic sublattice, the entropy change associated with the transitions is very small resulting in the absence of specific heat anomalies. Note that long-range magnetic order was found in BiFe_0.5_Sc_0.5_O_3_ [22] and LaMn_0.4_Ga_0.6_O_3_ [23] by neutron diffraction, thus, confirming that long-range magnetic ordering can occur in samples with highly diluted magnetic sublattices.

Magnetic properties of BiCr_0.3_Ga_0.7_O_3_ are principally different (figures 7 and 9(c)). BiCr_0.3_Ga_0.7_O_3_ demonstrates basically paramagnetic behaviour as can be clearly seen from the *χ*′ versus *T* curve and the absence of any anomalies on the *χ*″ versus *T* curves (figure 9(c)). BiCr_0.3_Ga_0.7_O_3_ still has a large Curie–Weiss temperature of about −73 K (table 1) indicating strong short-range AFM coupling between Cr^3+^ ions, but there should be no long-range ordering because *x* = 0.7 is above the percolation threshold of *x* = 0.69 for perovskite structures [24]; below the 31% concentration, a dopant cannot be linked in a continuous path throughout a crystal. We note that BiCr_0.3_Ga_0.7_O_3_ and other samples with *x* = 0.4–0.6 show a small divergence between the ZFC and FC curves below about 90 K; this feature most probably originates from trace amounts of a magnetic impurity (figures S5 and S6 of the ESI).

**Figure 10.**
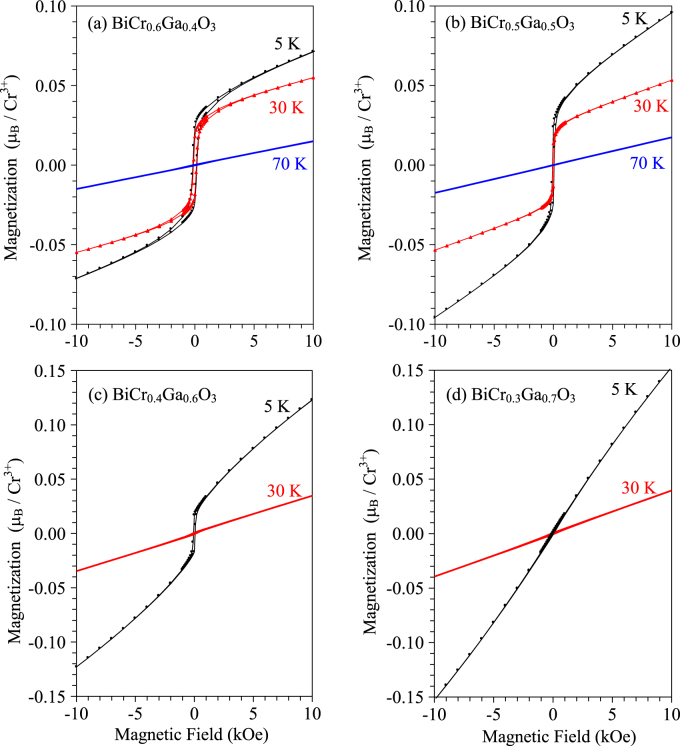
Isothermal magnetization curves of (a) BiCr_0.6_Ga_0.4_O_3_ (R3c) at 5, 30 and 70 K, (b) BiCr_0.5_Ga_0.5_O_3_ (R3c) at 5, 30 and 70 K, (c) BiCr_0.4_Ga_0.6_O_3_ (R3c) at 5 and 30 K and (d) BiCr_0.3_Ga_0.7_O_3_ (R3c) at 5 and 30 K.

Isothermal magnetization curves of BiCr_1−*x*_Ga_*x*_O_3_ with *x* = 0.4, 0.5, 0.6 and 0.7 are given in figure 10. They show that a weak ferromagnetic moment is developed below *T*_N_ in BiCr_1−*x*_Ga_*x*_O_3_ with *x* = 0.4, 0.5 and 0.6. In BiCr_0.6_Ga_0.4_O_3_, the magnetization reaches 0.17*μ*_B_/Cr at 5 K and 50 kOe; at 5 K, the remnant magnetization is 0.023*μ*_B_/Cr, and the coercitive field is about 180 Oe (figure S11 of the ESI).

**Figure 11.**
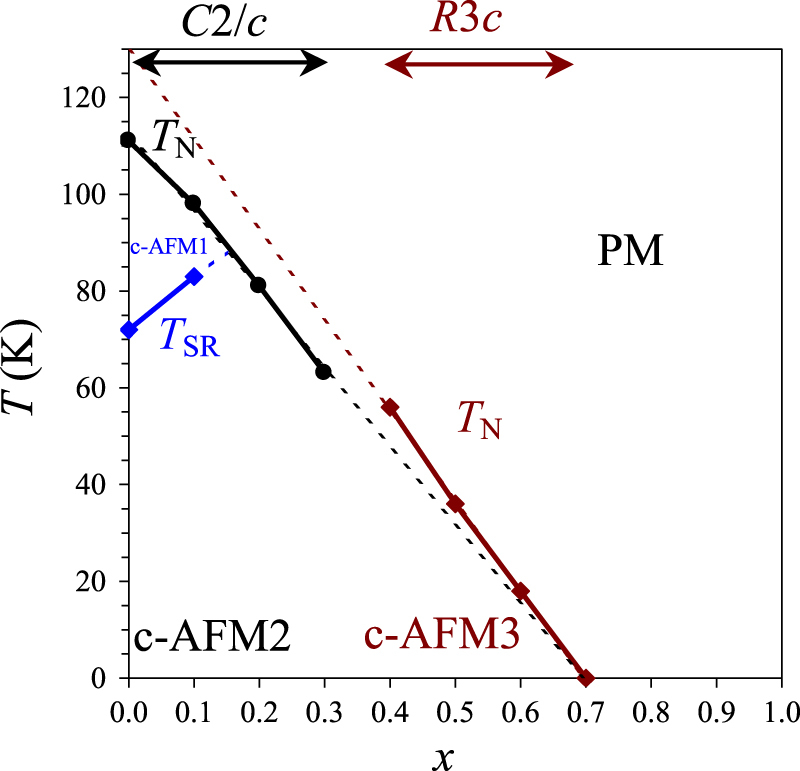
Magnetic phase diagram of BiCr_1−*x*_Ga_*x*_O_3_. *T*_N_ is the Néel temperature, *T*_SR_ is temperature of a spin-reorientation transition, PM is a paramagnetic phase and c-AFM is a canted antiferromagnetic phase.

The resulting magnetic phase diagram of BiCr_1−*x*_Ga_*x*_O_3_ is given in figure 11. The *T*_N_ gradually decreases with increasing *x*, as one would expect because of the dilution of the magnetic sublattice by a nonmagnetic ion, while *T*_SR_ increases. By the extrapolation, the *T*_N_ and *T*_SR_ should merge near *x* = 0.15. There is almost linear dependence of *T*_N_ on *x* in the compositional ranges of 0.0 ≤ *x* ≤ 0.3 and 0.4 ≤ *x* ≤ 0.7. In both ranges, *T*_N_ vanishes near *x* = 0.7 by the extrapolation, that is, near the percolation threshold. From the extrapolation, we can also estimate that *T*_N_ should be about 130 K for a hypothetical R3c phase of BiCrO_3_, which was studied theoretically in some papers [16, 25]; the theoretically estimated *T*_N_ is about 80–120 K [25]. The G-type AFM structure should realize in BiCrO_3_-based perovskites as predicted in many theoretical papers [15, 16, 25] and found experimentally [11–13]. However, spin canting mechanisms might be different depending on the symmetry; this is why different regions are marked as c-AFM1, c-AFM2 and c-AFM3 in figure 11. Spin canting is allowed by the symmetry in the C2/c and R3c structures and G-type magnetic arrangements.

In conclusion, we investigated magnetic properties of BiCr_1−*x*_Ga_*x*_O_3_ solid solutions. AFM order with weak ferromagnetism is observed below *T*_N_ = 54, 36 and 18 K in the samples with *x* = 0.4, 0.5 and 0.6, respectively, having a polar R3c structure. *T*_N_ decreases from 111 K in BiCrO_3_ to 98 K in BiCr_0.9_Ga_0.1_O_3_, and the spin-reorientation transition temperature increases from 72 K in BiCrO_3_ to 83 K in BiCr_0.9_Ga_0.1_O_3_, having C2/c symmetry. A magnetic phase diagram of BiCr_1−*x*_Ga_*x*_O_3_ is constructed.
